# A mutation-independent CRISPR-Cas9–mediated gene targeting approach to treat a murine model of ornithine transcarbamylase deficiency

**DOI:** 10.1126/sciadv.aax5701

**Published:** 2020-02-12

**Authors:** Lili Wang, Yang Yang, Camilo Breton, Peter Bell, Mingyao Li, Jia Zhang, Yan Che, Alexei Saveliev, Zhenning He, John White, Caitlin Latshaw, Chenyu Xu, Deirdre McMenamin, Hongwei Yu, Hiroki Morizono, Mark L. Batshaw, James M. Wilson

**Affiliations:** 1 Gene Therapy Program, Department of Medicine, University of Pennsylvania, Philadelphia, PA 19104, USA.; 2 State Key Laboratory of Biotherapy and Cancer Center, West China Hospital, Sichuan University, and Collaborative Innovation Center for Biotherapy, Chengdu, Sichuan, China.; 3 Department of Biostatistics and Epidemiology, University of Pennsylvania, Philadelphia, PA 19104, USA.; 4 Center for Genetic Medicine Research, Children’s Research Institute, Children’s National Hospital, Washington, DC 20010, USA.

## Abstract

Ornithine transcarbamylase (OTC) deficiency is an X-linked urea cycle disorder associated with high mortality. Although a promising treatment for late-onset OTC deficiency, adeno-associated virus (AAV) neonatal gene therapy would only provide short-term therapeutic effects as the non-integrated genome gets lost during hepatocyte proliferation. CRISPR-Cas9-mediated homology-directed repair can correct a G-to-A mutation in 10% of OTC alleles in the livers of newborn OTC *spf^ash^* mice. However, an editing vector able to correct one mutation would not be applicable for patients carrying different OTC mutations, plus expression would not be fast enough to treat a hyperammonemia crisis. Here, we describe a dual-AAV vector system that accomplishes rapid short-term expression from a non-integrated minigene and long-term expression from the site-specific integration of this minigene without any selective growth advantage for OTC-positive cells in newborns. This CRISPR-Cas9 gene-targeting approach may be applicable to all patients with OTC deficiency, irrespective of mutation and/or clinical state.

## Teaser

AAV- and CRISPR-Cas9–mediated gene targeting in neonatal mice led to clinical benefits in a mouse model of OTC deficiency.

## INTRODUCTION

Ornithine transcarbamylase (OTC) deficiency (OTCD) is an X-linked recessive disorder that accounts for nearly half of all inborn errors of the urea cycle (*1*). Severe OTCD in the neonatal period can result in hyperammonemic coma, which can rapidly become fatal without treatment (*2*). Current therapies include dialysis, the use of alternate nitrogen clearance pathways, and liver transplantation for severely affected patients; however, the mortality rate is still high (*3*).

Adeno-associated virus (AAV) vector–based gene therapy could provide an alternative to current treatment options. Over the past few years, AAV gene therapy has shown promising results in clinical trials for several diseases (*4*–*7*). Recently, the Food and Drug Administration approved the first AAV gene augmentation therapy for an inherited disease (*8*). An AAV8 vector that we have developed is currently being evaluated in a clinical trial for adult patients with OTCD (*9*). For patients with an early-onset form of OTCD, treatment in the early stage would be desirable. However, AAV-mediated, liver-directed neonatal gene therapy would only achieve short-term effects (*10*–*13*). Because of the nonintegrating nature of the AAV vector, most of the vector genome would be lost during hepatocyte proliferation. We hypothesize that directed integration of the OTC transgene into the host genome by genome editing could solve this problem.

Targeted genome editing is the holy grail of gene therapy (*14*). Pioneered by zinc finger nucleases (ZFNs), which rely on protein-DNA binding, genome editing initially entered the clinic with an ex vivo approach (*15*) and more recently in vivo using an AAV vector to target the liver of patients with hemophilia B or mucopolysaccharidosis I (*16*, *17*). The discovery and development of CRISPR-Cas9 as a genome editing technology have provided a relatively simple method for site-specific genome modifications, owing to its unique mechanism of RNA-mediated DNA binding (*18*). Upon generation of site-specific double-stranded breaks (DSBs), nonhomologous end joining (NHEJ) creates insertions and deletions (indels); when a donor DNA template is present, homology-directed repair (HDR) incorporates the DNA sequence on the donor template into the endogenous locus. Given its high transduction efficiency in many tissues, AAV vector has been used as an efficient vehicle to deliver the nucleases and/or donor template for in vivo genome editing applications (*19*–*24*).

We recently developed a dual AAV vector approach for in vivo delivery of three key components of the CRISPR-Cas9 system: Cas9 enzyme from *Staphylococcus aureus* (SaCas9), a single-guide RNA (sgRNA) to a sequence in the murine OTC gene, and a donor template to drive HDR (*25*). We demonstrated HDR-based correction of a G-to-A mutation in 10% of OTC alleles in the livers of newborn *spf^ash^* mice, which provide a model of chronic hyperammonemia, and clinical benefits following in vivo genome editing. However, this vector, which was developed for a specific mutation, would not be applicable for patients with mutations elsewhere in the OTC gene. Like most monogenic diseases, OTCD is caused by >300 different mutations scattered throughout the gene rather than a single predominant mutation (*27*). Moreover, our previous mutation correction approach is not only ineffective in adult *spf^ash^* mice but also causes lethality in *spf^ash^* mice treated as adults due to the loss of residual OTC expression caused by large deletions extending to exon 4 of the mouse OTC (mOTC) gene (*25*).

In this study, we aimed to develop a mutation-independent CRISPR-Cas9–mediated gene targeting approach, in which the vector system could be applied to most patients with a specific disease, in this instance, OTCD. We report that a single injection of a dual AAV8 vector system in neonatal mice achieves transient, high-level expression from the unintegrated transgene that could be useful in treating the acute neonatal crisis. Genome editing–directed integration of the transgene results in efficient, sustained, and clinically beneficial gene targeting in liver in the absence of any selective growth advantage for OTC-positive cells.

## RESULTS

### Development of a dual AAV vector system for CRISPR-Cas9–mediated gene targeting

**Fig. 1:**
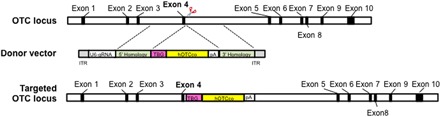
In vivo gene targeting of the OTC locus in the OTC *spf^ash^* mouse liver by AAV.SaCas9. Schematic diagrams of the mouse OTC locus showing the SaCas9 target site located in intron 4, the AAV donor vector that contains U6-sgRNA1 and TBG-hOTCco-pA cassettes flanked by homology arms, and the modified mouse OTC locus after homologous recombination. ITR, inverted terminal repeat.

To develop a broadly applicable genome editing vector for OTCD, we constructed a new AAV8 donor vector that contains (i) an sgRNA driven by the U6 promoter to target intron 4 of the mOTC locus (*25*) and (ii) a fully functional minigene expressing codon-optimized human OTC (hOTCco) driven by a liver-specific thyroxine binding globulin (TBG) promoter (TBG.hOTCco.pA) flanked by 0.9-kb homology arms on each side (referred to as AAV8.targeted donor; Fig. 1). The untargeted control donor vector contains all components except for the 20-nucleotide target sequence (referred to as AAV8.untargeted donor). Following CRISPR-Cas9–mediated HDR, the transgene cassette (referred to as the hOTCco minigene) should be inserted into intron 4 of the mOTC locus (Fig. 1). We selected intron 4 as the site of integration because it was used to correct the mutation in the *spf^ash^* mice, providing a direct comparison of the two approaches independent of the efficiency and site of the on-target DSBs (*25*).

### In vivo gene targeting of the OTC locus in the OTC *spf^ash^* mouse liver by AAV.SaCas9

**Fig. 2:**
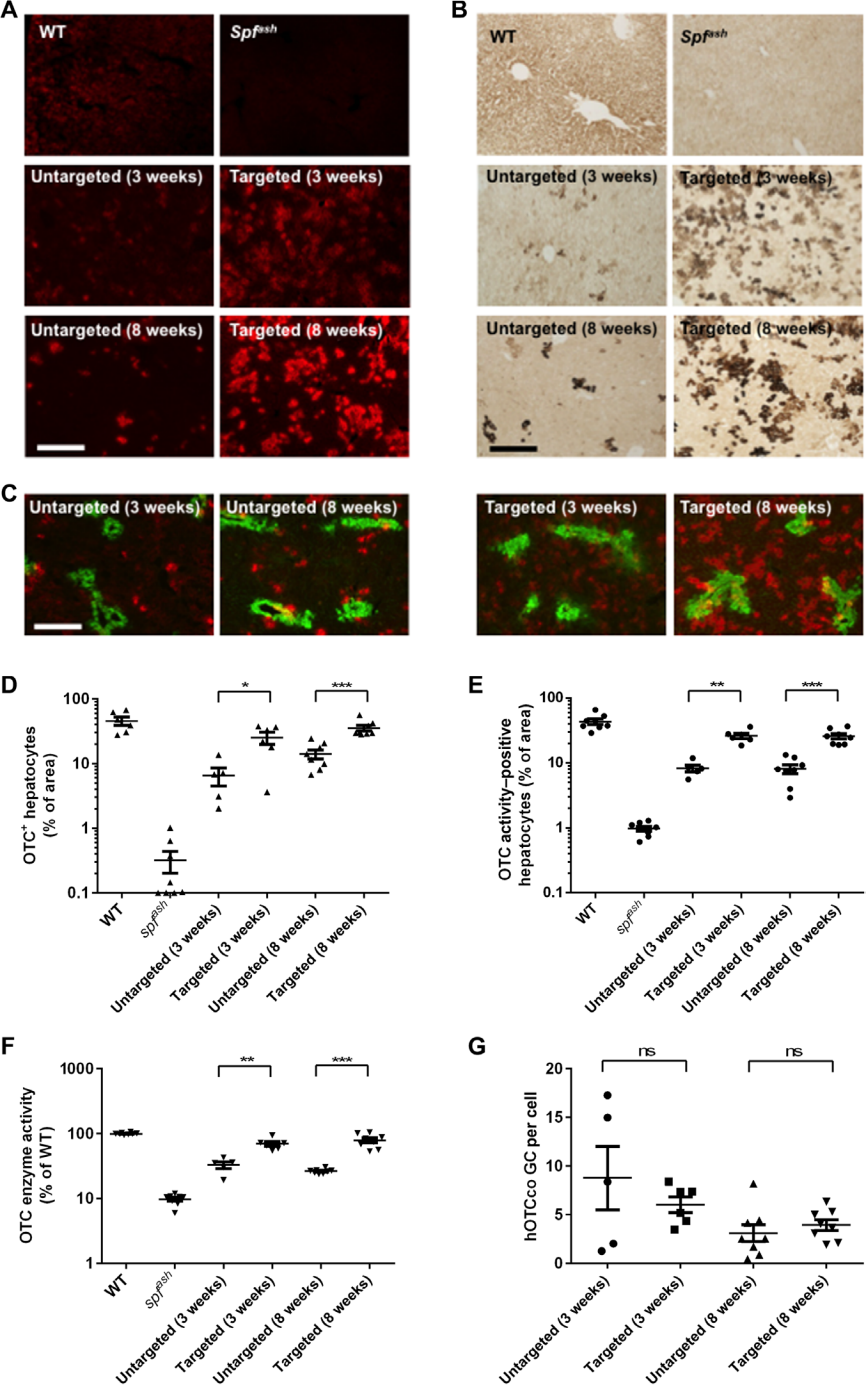
Efficient and sustained expression of OTC in the liver of *spf^ash^* mice treated as newborns with AAV8.SaCas9-mediated gene targeting. AAV8.SaCas9 (5 × 10^10^ GC per pup) and AAV8.sgRNA1.hOTCco donor (5 × 10^11^ GC per pup) were administrated to p2 *spf^ash^* pups via the temporal vein. *spf^ash^* mice were euthanized at 3 (targeted, 3 weeks; *n* = 6) or 8 weeks (targeted, 8 weeks; *n* = 8) after treatment. Untargeted *spf*^*as*h^ mice received AAV8.SaCas9 (5 × 10^10^ GC per pup) and AAV8.control.hOTCco donor (5 × 10^11^ GC per pup) at p2, and livers were harvested at 3 (untargeted, 3 weeks; *n* = 5) or 8 weeks (untargeted, 8 weeks; *n* = 8] after treatment. Untreated WT (*n* = 8) and *spf^ash^* mice (*n* = 8) were included as controls. (**A**) Immunofluorescence staining with antibodies against OTC on liver sections from *spf^ash^* mice treated with the dual AAV vectors for CRISPR-SaCas9–mediated gene targeting. Stained areas in the targeted groups typically represent clusters of OTC-expressing hepatocytes. Scale bar, 200 μm. (**B**) Histochemical staining of OTC enzyme activity on liver sections from *spf^ash^* mice treated with the dual AAV vectors for CRISPR-SaCas9–mediated gene targeting. Scale bar, 200 μm. (**C**) Double immunofluorescence staining with antibodies against OTC (red) and glutamine synthetase (green), which is a marker of central veins. Scale bar, 200 μm. (**D**) Quantification of OTC-expressing cells based on the percentage of area on liver sections expressing OTC by immunostaining as presented in (A). (**E**) Quantification of hepatocytes expressing functional OTC based on the percentage of area on liver sections expressing OTC as presented in (B). (**F**) OTC enzyme activity in the liver homogenates of *spf^ash^* mice at 3 and 8 weeks following dual vector treatment. (**G**) Quantification of hOTCco donor vector genome in the liver by quantitative PCR. ns, not statistically significant. *P < 0.05, **P < 0.01, ***P < 0.001, Mann-Whitney test.

To determine the in vivo gene targeting efficiency, we coinjected AAV8.SaCas9 [5 × 10^10^ genome copies (GC) per pup] and AAV8.targeted donor or AAV8.untargeted donor (5 × 10^11^ GC per pup) vectors into postnatal day 2 (p2) *spf^ash^* male pups via the temporal vein. We harvested liver samples at 3 and 8 weeks after vector injection for immunohistochemistry of OTC and histochemical staining of OTC enzyme activity (Fig. 2, A to C). Mice treated with the targeted vector (referred to as targeted mice) showed 25 and 35% of OTC-expressing hepatocytes at 3 and 8 weeks, respectively; this was four- and threefold higher than the mice treated with the untargeted vector (referred to as untargeted mice) at the same time points (Fig. 2D). Treated animals showed clusters of OTC-expressing cells scattered in the liver, including regions around the central vein where urea cycle enzymes are not normally expressed (Fig. 2C). Histochemical staining of OTC enzyme activity in the targeted mice at both 3 and 8 weeks showed expression of functional OTC in 26% of hepatocytes, which was threefold higher than that in the untargeted mice (Fig. 2E). At both time points, most OTC-positive hepatocytes were located in clusters scattered throughout all portions of the liver in the targeted mice, consistent with integration followed by clonal expansion in the context of a growing liver. Direct measurements of OTC enzyme activity from liver homogenates obtained from the targeted mice at 3 and 8 weeks showed 70 and 79% of wild-type (WT) levels, respectively, which were two- and threefold higher than the untargeted mice at the same time points (Fig. 2F). The OTC activity levels measured in the liver homogenates were about threefold higher than the percentage of OTC-positive hepatocytes by histochemical staining of OTC enzyme activity (Fig. 2E), suggesting that edited hepatocytes express levels of OTC higher than the endogenous gene. This may be due to the codon optimization of the hOTCco complementary DNA and the use of a strong liver-specific TBG promoter in the minigene. The OTC expression levels in individual hepatocytes from the hOTCco minigene were higher than those in hepatocytes in WT mice, as demonstrated by immunohistochemistry (Fig. 2, A and B).

We also measured hOTCco copies in the liver by quantitative polymerase chain reaction (PCR). At 3 weeks after vector treatment, we detected seven to eight copies of hOTCco per diploid genome in both untargeted and targeted mice (Fig. 2G). At 8 weeks after vector treatment, hOTCco copies decreased to two copies per diploid genome in untargeted mice and five copies in targeted mice, yet the difference between the targeted and untargeted groups was not statistically significant (Fig. 2G). Some hOTCco donor vector DNA (untargeted or targeted) may exist in the cells as episomal concatemers or have been randomly integrated into the host genome.

### Clinical benefits evaluated by a high-protein diet challenge

**Fig. 3:**
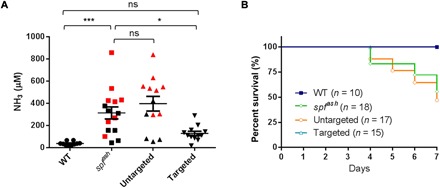
Functional improvement following high-protein diet challenge in *spf^ash^* mice treated with the dual vectors for gene targeting. Seven weeks following neonatal treatment with the dual AAV vectors, mice were given high-protein diet for 7 days. (**A**) Plasma ammonia levels were measured 7 days after the high-protein diet. Plasma ammonia levels in WT mice (*n* = 10) and AAV8.SaCas9 + AAV8.sgRNA1.hOTCco donor-treated *spf^ash^* mice (*n* = 12) were significantly lower than in untreated *spf^ash^* mice (*n* = 15) after a 7-day high-protein diet. Red squares indicate samples obtained from moribund untreated *spf^ash^* mice before the scheduled day 7 bleed; red triangles indicate sample obtained from a moribund *spf^ash^* mice treated with untargeted vector (AAV8.control.hOTCco donor with no sgRNA1, *n* = 13) before the scheduled day 7 bleed. ns, not statistically different; *P < 0.05, ***P < 0.001, one-way analysis of variance (ANOVA), Dunnett’s test. (**B**) Survival curves in control or dual AAV vector–treated *spf^ash^* mice after a 7-day course of high-protein diet. Untreated *spf^ash^* mice (*n* = 18) or *spf^ash^* mice treated with untargeted vectors (AAV8.control.hOTCco donor, *n* = 17) started to die 4 days after the high-protein diet. All WT (*n* = 10) and AAV8.SaCas9 + AAV8.sgRNA1.hOTCco donor-treated *spf^ash^* mice (*n* = 15) survived. *P* < 0.05, log-rank test.

To further assess the impact of gene targeting on the clinical manifestations of OTCD, we evaluated the tolerance of *spf^ash^* mice to a 7-day course of a high-protein diet at 7 weeks postneonatal vector administration. We included WT littermates, untreated *spf^ash^* mice, and untargeted *spf^ash^* mice as controls. At the end of the course of the high-protein diet, we found that plasma ammonia was elevated from 37 ± 5 μM (*n* = 10) in WT controls to 314 ± 55 μM (*n* = 15) in the *spf^ash^* controls (*P* < 0.001) (Fig. 3A), consistent with our previous finding. We observed substantial variations in plasma ammonia levels in the untreated *spf^ash^* mice, consistent with findings in patients with OTCD who show considerable fluctuations in ammonia over relatively short periods of time (*26*). We did not observe a significant difference between untreated *spf^ash^* mice and untargeted *spf^ash^* mice (*n* = 13; Fig. 3A), which indicates that the residual hOTC expression in the untargeted mice was not sufficient to achieve clinical benefits. In contrast, we observed a statistically significant 60% reduction in ammonia levels in targeted mice (*n* = 12) as compared to untreated *spf^ash^* mice (*P* < 0.05; Fig. 3A), slightly more efficient than what we achieved by our previous gene correction approach (40% reduction) (*25*). The plasma ammonia levels in the targeted group were reduced with respect to untreated *spf^ash^* animals and did not statistically differ from those in WT mice showing complete correction. All WT mice (*n* = 10) and targeted mice (*n* = 15) survived the 7-day course of a high-protein challenge, whereas 27% of untreated *spf^ash^* mice (*n* = 18) and 35% of untargeted *spf^ash^* mice (*n* = 17) developed clinical signs of hyperammonemia, became lethargic, and had to be euthanized before the end of the 7-day course (Fig. 3B).

### On-target indel frequency and HDR-mediated gene targeting efficiency

**Fig. 4:**
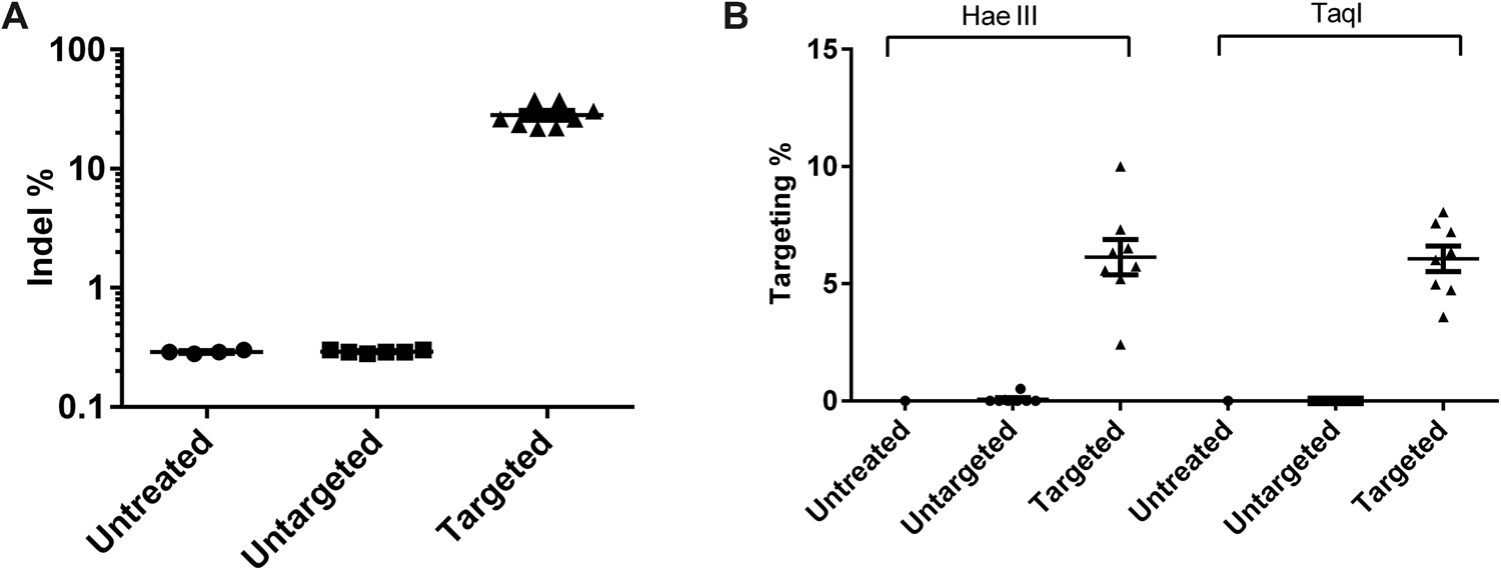
Indel and HDR-mediated gene targeting efficiency analyses. Liver DNA was isolated from *spf^ash^* mice 8 weeks after neonatal treatment with the dual gene-targeting vectors (*n* = 8) or untargeted vectors (*n* = 8). DNA from an untreated *spf^ash^* mouse served as control. (**A**) Indel analysis on the targeted mOTC locus by deep sequencing. (**B**) HDR-mediated gene targeting efficiency analysis by LMU-PCR following digestion with Hae III or TaqI (see fig. S2). Means ± SEM are shown.

To assess the on-target editing efficiency at the mOTC intron 4, we analyzed on-target indel frequency in DNA isolated from *spf^ash^* mice 8 weeks after vector treatment by deep sequencing of PCR amplicons of the targeted mOTC locus. The targeted mice had a mean indel frequency of 28% (22 to 38%, *n* = 8) (Fig. 4A). This was similar to the indel frequency that we observed with our previous gene correction approach using the same guide RNA (*25*). The untargeted mice had background levels at 0.3%, similar to the untreated mice (Fig. 4A).

To estimate the HDR-mediated gene targeting efficiency, we first digested the genomic DNA with Hae III or TaqI, which has recognition sites in the homology arms and in the donor vector (fig. S1, A and B), followed by ligation-mediated PCR coupled with unique molecular indices (LMU-PCR), which generate relatively similar sizes of PCR amplicons from untargeted (1.21 kb) and targeted OTC loci (1.09 and 0.99 kb for TaqI- and Hae III–digested samples, respectively) (fig. S1, C and D). Following deep sequencing of the nested PCR amplicons, we analyzed the mapped reads to calculate the ratio of reads containing the expected hOTCco minigene sequence and total mapped reads and defined it as HDR-mediated gene targeting efficiency. The targeted mice showed a mean targeting efficiency of 6% by both Hae III and TaqI assays (2 to 10% for Hae III, 4 to 8% for TaqI; *n* = 8; Fig. 4B). The untargeted mice (*n* = 8) showed undetectable (TaqI assay) or background levels (0.07%; Hae III assay) (Fig. 4B).

A more detailed analysis of the sequences obtained by LMU-PCR revealed a more complex pattern in the nature of the target region after genome editing. In addition to the sequences corresponding to parental genomic DNA and HDR-mediated insertion of the transgene, we found sequences mapping to different elements of the AAV vector such as the promoter, polyA, transgene, and inverted terminal repeats (ITRs) (fig. S2).

### Comparison of neonatal OTC gene therapy and CRISPR-Cas9–mediated gene correction and gene targeting

**Fig. 5:**
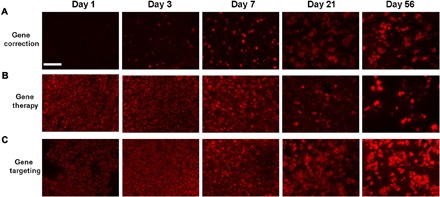
Time course of OTC expression in liver by neonatal gene therapy, CRISPR-Cas9–mediated gene correction, or gene targeting. p2 *spf^ash^* mice received temporal vein injection of AAV8.SaCas9 (5 × 10^10^ GC per pup) and 5 × 10^11^ GC per pup of AAV8.sRNA1.donor vector for CRISPR-Cas9–mediated gene correction (**A**), AAV8.control.hOTCco donor (equivalent of a gene therapy vector, **B**), or AAV8.sgRNA1.hOTCco donor for CRISPR-Cas9–mediated gene targeting (**C**). Liver samples were harvested at 1, 3, 7, 21, and 56 days after vector injection for immunostaining with an OTC antibody. Representative pictures at each time point are shown. Scale bar, 200 μm.

To compare the gene targeting approach with our previously developed CRISPR-Cas9–mediated gene correction strategy and neonatal gene therapy, we treated p2 *spf^ash^* mice with AAV8.SaCas9 in combination with vectors for gene targeting (AAV8.sgRNA1.hOTCco donor), gene correction of the *spf^ash^* mutation (AAV8.sRNA1.donor vector), or gene therapy (AAV8.control.hOTCco donor). We harvested liver samples at 1, 3, 7, 21, and 56 days after vector injection to evaluate the kinetics of OTC expression by immunostaining. For the CRISPR-Cas9–mediated gene correction approach, isolated OTC-expressing cells started to appear at day 3 after vector treatment at a low frequency and increased over time (Fig. 5A). *Spf^ash^* mice treated with the gene therapy vector (Fig. 5B) or gene-targeting vector (Fig. 5C) showed OTC-expressing cells as early as day 1 with a rapid increase to virtually all cells expressing OTC by day 7. These two groups, however, diverged between days 21 and 56, with the mice treated with the gene therapy vector showing decreased OTC-expressing cells (Fig. 5B) and the mice treated with the gene-targeting vector showing clusters of OTC-positive cells (Fig. 5C). Therefore, because of the slow kinetics of OTC expression, the gene correction approach would not be suitable to treat patients with OTC during the acute phase of hyperammonemia, although it would have long-lasting effects after the initial phase after treatment. Conversely, neonatal gene therapy with AAV alone functions quickly during the acute phase of hyperammonemia, but its therapeutic effects attenuate following hepatocyte proliferation. The gene targeting approach, however, would have both therapeutic benefits at an early phase and sustained efficacy through hepatocyte proliferation in the absence of any selective growth advantage for OTC-positive cells.

## DISCUSSION

Using a highly efficient AAV delivery platform together with potent and specific guide RNAs for CRISPR-Cas9, we and others have demonstrated efficient in vivo genome editing in mouse models (*25*). Following cleavage by endonuclease, HDR is generally a less efficient pathway compared to NHEJ, which creates gene-disabling indels. AAV vector has exhibited advantages as an efficient vehicle to deliver donor DNA both in vitro and in vivo. We previously demonstrated successful correction of a G-to-A mutation in 10% of OTC alleles in the liver of newborn OTC *spf^ash^* mice by a CRISPR-Cas9–mediated HDR approach (*25*). However, this approach cannot benefit all OTC-deficient patients because disease-causing mutations and large deletions are found scattered at approximately 320 different positions throughout the *OTC* gene (*27*). The HDR-mediated gene-targeting approach described in the current study could be broadly applied to all patients carrying mutations in the same causal gene, similar to gene replacement therapy, as long as they do not carry polymorphisms at the guide RNA target site. In the current proof-of-concept study, we used the same guide RNA as our previous study to target intron 4 of the murine *OTC* gene, 47 base pairs (bp) downstream of the *spf^ash^* mutation. As a result, we could compare the efficiency of the two approaches without the complication of having different efficiencies by different guide RNAs. One of the advantages of gene targeting of a minigene is the flexibility of the targeting site. The gene targeting site can be explored in safe harbors containing protospacer sequences for highly efficient and specific guide RNAs. In contrast, for the gene correction approach, the selection of guide RNA is limited by the availability of protospacer adjacent motif sequences adjacent to the mutation. Among the limited choices of guide RNAs, a guide RNA with both sufficient efficiency and specificity may not exist.

For many genetic diseases that present clinically in the neonatal period with lethal effects, such as urea cycle disorders, early treatment and sustained therapeutic efficacy are essential. We compared three approaches: neonatal gene therapy with AAV expressing an OTC minigene alone, CRISPR-Cas9–mediated gene correction, and CRISPR-Cas9–mediated targeted integration of a therapeutic transgene cassette. The latter showed the advantages of neonatal gene therapy during the early phase combined with long-term benefits from the integrated transgene cassette due to genome editing (Fig. 5). Unlike the gene correction approach, not all OTC-expressing hepatocytes were derived from HDR-mediated genome editing. The source of OTC expression in this system could be multifactorial, being derived from the episomal donor vector genome that persists despite dilution from proliferating cells or a randomly integrated vector genome as seen in mice treated with the control donor vector (Fig. 5B) in addition to site-specific integration of the minigene. In mice treated with the gene-targeting vector, most of the OTC expression at early time points was derived from episomal vector DNA, similar to neonatal AAV gene therapy. With the high dose of donor vector used in this study, the OTC expression levels in the early phase (i.e., the first week) are likely to be over the normal levels, which do not have untoward effects. At later time points, most of the OTC expression came from targeted integration of the human OTC minigene. Also similar to the gene therapy approach and as predicted for endogenous OTC, OTC-expressing cells with the gene-targeting approach were scattered in the liver, including around central veins (Fig. 2C). This was in contrast to the gene correction approach, in which OTC-expressing cells were localized within all portions of the portal axis except around the central veins (*25*). OTC-expressing cells in the pericentral areas would have no impact on ureagenesis, as the urea cycle is least active in these areas. Although our dual vector gene-targeting approach achieved clinical benefits in OTC *spf^ash^* mice treated as neonates, it should be noted that the vector dose used in the gene-target study is much higher than those used in AAV gene therapy studies, which, in most cases, only involve a single vector.

Because of the large homology arms (~900 bp at each end) of the donor vector, there is no straightforward way to measure HDR-mediated gene targeting efficiency. Therefore, we developed a method, called LMU-PCR, to quantify the levels of insertion of the hOTCco minigene. Assays with two different restriction enzymes showed a similar targeting efficiency of 6% (Fig. 4B). Detailed analysis of the sequences obtained by LMU-PCR revealed a complex pattern of genome structure around the target region (fig. S2). Besides the sequences corresponding to parental genomic DNA and HDR-mediated insertion of the hOTCco minigene, different parts of the AAV vector sequences were found inserted into the DSB, including partial portions of the promoter, polyA, transgene, and AAV ITRs. Some of these insertions may be too large to be captured by PCR amplicon next-generation sequencing (NGS); therefore, PCR amplicon NGS likely underestimates the true indel frequencies. The subcategories of insertions detected by this method can be influenced by the location of the restriction enzyme sites. We detected more ITR sequences in Hae III–digested versus TaqI-digested samples, likely because Hae III cuts three times in the ITR and TaqI does not cut within this region. The hairpin structure of the AAV ITR is known to impede PCR amplification (*28*). When delivered by AAV vectors, insertion of AAV sequences into the Cas9 cleavage site is expected, as AAV vector sequences can be integrated into the genome after induction of DSBs in the cell genome (*29*). Furthermore, AAV ITR sequences were detected after treatment of cells with a ZFN targeting the AAVS1 locus (*30*). These studies have shown that insertion of AAV sequences by NHEJ is an expected secondary effect of any AAV-delivered genome editing approach.

We cannot determine the extent of the AAV sequences integrated into the target region because of the limitations of the NGS amplicons. However, it is possible that some hOTC expression is derived from the donor vector inserted by NHEJ rather than HDR, as the donor vector contains promoter and polyA signals. NHEJ-mediated integration of the gene-targeting vector has been reported in previous studies using ZFNs (*31*, *32*). Last, some hOTC expression is likely due to the episomal donor vector DNA or randomly integrated donor vector, as seen in the untargeted mice.

Before CRISPR-Cas9–mediated gene targeting, AAV/ZFN-mediated in vivo genome targeting in the liver demonstrated sustained and therapeutic levels of coagulation factor IX in both neonatal and adult mice (*31*–*34*). To take advantage of the strong albumin promoter, researchers developed ZFN to target the albumin locus and achieved high levels of gene expression for multiple transgenes (*32*). Encouraged by the preclinical data, clinical trials in patients with hemophilia B or mucopolysaccharidosis I (*16*, *17*) have started. Although this approach has worked well for secreted proteins, the targeting efficiency at ~0.5% is likely too low to be effective for nonsecreted proteins such as enzymes of the urea cycle that require broad expression in a large number of hepatocytes.

In conclusion, we have demonstrated the therapeutic effect of AAV-delivered, CRISPR-Cas9–mediated gene targeting in a mouse model of OTCD. In the absence of any selective growth advantage for OTC-positive cells, a single injection of dual AAV gene-targeting vectors in neonatal mice achieved robust and sustained expression of OTC that was clinically beneficial. This genome editing strategy is not mutation position specific and can be broadly applied to all patients with the same disease. This strategy can also be adapted to other hereditary disorders of the liver.

## MATERIALS AND METHODS

### Study design

#### Sample size

Test and control vectors were evaluated in at least five mice per group at each time point to ensure reproducibility. Sample sizes are noted in figure legends.

#### Outliers

All data are presented.

#### Selection of endpoints

We chose 3 and 8 weeks after dosing in neonatal mice as relative short- and long-term time points, respectively, to evaluate OTC expression and gene targeting efficiency.

#### Replicates

Each vector group was evaluated in multiple litters of male *spf^ash^* pups. Transduction efficiency in each mouse was analyzed on at least five images. Plasma NH_3_ was assayed in duplicate. Indel and HDR analysis on each sample was performed once.

#### Research objectives

This study aimed to develop a mutation-independent CRISPR-Cas9–mediated gene targeting approach in which the vector system could be applied to most patients with OTCD.

#### Research subjects

Newborn (p2) male *spf^ash^* mouse pups were the subjects of this study. Untreated WT and *spf^ash^* hemizygous mice served as controls.

#### Experimental design

Pups received a temporal vein injection of a mixture of two vectors at the intended doses for each with a volume of 50 μl. Mice were euthanized at various time points after vector treatment, and liver samples were harvested for analyses. Mice were genotyped at weaning or at the time of necropsy to confirm genotype. For testing the efficacy of *OTC* gene targeting, a high-protein diet (40% protein; Animal Specialties and Provisions, Quakertown, PA) was given to 7-week-old mice for 7 days. After this time, plasma was collected for measurement of plasma NH_3_ using an Ammonia Assay Kit (Sigma-Aldrich, St. Louis, MO).

#### Randomization

Note that the entire litter of newborn male pups was injected with either the targeting or control vectors, and no specific randomization method was used.

#### Blinding

The following assays were performed in a blinded fashion, in which the investigator was unaware of the nature of the vectors or vector dose: vector injection, OTC immunostaining, OTC enzyme activity staining and quantification, OTC enzyme activity assay, and vector GC analysis.

### Plasmid construction

The AAV *OTC* gene-targeting vector contains the U6-OTC sgRNA1 cassette and the TBG.hOTCco.pA cassette flanked by 0.9-kb homology arms on each side (Fig. 1). This vector was generated by cloning of the TBG.hOTCco.pA cassette from pAAVss.TBG.hOTCco (*35*) into the Mfe I site of the pAAV.sgRNA1.donor, which was previously constructed for gene correction of the OTC *spf^ash^* mutation (*25*). The “untargeted” AAV.control.targeting donor differs from the “targeted” AAV.sgRNA1.TBG.hOTCco.pA.donor by the lack of the protospacer sequence from the U6-OTC sgRNA1 cassette. All plasmid constructs were verified by sequencing. The SaCas9 expression vector AAV.TBG.SaCas9 has been previously described (*25*).

### AAV vector production

All AAV8 vectors were produced by the Penn Vector Core at the University of Pennsylvania as previously described (*36*). The genome titer (GC ml^−1^) of AAV vectors was determined by quantitative PCR. All vectors used in this study passed an endotoxin assay using the QCL-1000 Chromogenic LAL test kit (Cambrex Bio Science).

### Animal studies

*spf^ash^* mice were maintained in an Association for Assessment and Accreditation of Laboratory Animal Care–accredited and Public Health Service–assured facility at the University of Pennsylvania, as described previously (*37*). All animal procedures were performed in accordance with protocols approved by the Institutional Animal Care and Use Committee of the University of Pennsylvania. Mating cages were monitored daily for births.

### OTC enzyme activity staining and OTC immunostaining

Sliced liver tissue (2 mm) was fixed, embedded, sectioned (9 μm), and mounted onto slides for histochemical staining of OTC enzyme activity, as previously described (*37*). Immunofluorescence for OTC and glutamine synthetase expression was performed on frozen liver sections, as previously described (*37*). Quantification of percentages of OTC-expressing hepatocytes was performed as previously described (*25*).

### OTC enzyme activity assay

OTC enzyme activity was assayed on liver homogenates as previously described (*25*).

### Vector GC analysis

Genomic DNA was isolated from liver and extracted using the QIAamp DNA mini kit (Qiagen). Vector genomes were quantified by real-time PCR using primers/probe set corresponding to hOTCco.

### In vivo on-target indel frequency analysis

On-target indel frequencies were evaluated on liver samples collected at 8 weeks following vector administration by deep sequencing on PCR amplicons using primers and methods described previously (*24*, *25*). Libraries were made from 250 ng of the 433-bp PCR products using the NEBNext Ultra II DNA Library Prep Kit for Illumina (New England BioLabs) following the manufacturer’s instructions. Individual libraries were analyzed for quality and size using a high-resolution cartridge in the QIAxcel advanced system (Qiagen); library concentrations were measured by the PicoGreen assay (Thermo Fisher Scientific, Waltham, MA) before library pooling at equal molarity. The pooled library was subsequently size selected from 300 to 800 bp using an E-Gel Electrophoresis system (Thermo Fisher Scientific) and a QIAquick Gel Extraction kit (Qiagen). The final pooled and purified NGS library was quantified by a Qubit 3.0 Fluorometer, denatured, and diluted to 8 pM according to Illumina’s instructions. To increase library diversity for on-target indel analysis, 10 to 15% PhiX and the same percentage of an irrelevant, barcoded NGS library made from plasmid DNA were supplemented into the final amplicon-library pool before being loaded onto a MiSeq reagent cartridge. Sequencing was then performed using an Illumina MiSeq Reagent V2 kit (250-bp pair end; Illumina) to enable successful read merging for downstream data analysis.

Paired-end read pairs were assembled using PEAR46. The merged reads were aligned to the reference sequence with Burrows-Wheeler aligner (BWA) maximal exact match (MEM) (http://bio-bwa.sourceforge.net/), and the mapped reads were filtered to have mapping quality scores of at least 20. To identify indels, the target window was defined as a region that is within 20 bp upstream or downstream of the predicted cleavage site. We counted the total number of reads that mapped to the target window and the number of reads that contained indels. The indel frequency was calculated as the number of indel-containing reads divided by the total number of reads mapped to the target window.

### Gene targeting efficiency analysis

HDR-mediated integration of the hOTCco cassette into the mouse OTC locus was quantified by LMU-PCR, as previously described (*39*). Briefly, 1 μg of genomic DNA was digested with 20 U of Hae III or TaqI for 4 hours at 37°C (for Hae III) or 65°C (for TaqI). Digested DNA was purified with Agencourt AMPure XP beads (Beckman Coulter, Brea, CA) at a ratio of 2× and eluted in 20 μl of elution buffer (Qiagen). Purified DNA was quantified using the Quant-It PicoGreen dsDNA assay (Thermo Fisher Scientific, Waltham, MA). A total of 180 ng of purified DNA was end-repaired and ligated to Y-adapters (see table S1), containing unique molecular indexes to reduce PCR bias, as previously described (*40*). Ligated DNA was purified with AMPure XP beads at a ratio of 0.9× and eluted in 15 μl of elution buffer (Qiagen). DNA was amplified by touchdown PCR using Platinum Taq DNA polymerase (Thermo Fisher Scientific) and the primers P5_1 plus either left-OTC-F1 (for Hae III–digested DNA) or right-OTC-F1 (for TaqI-digested DNA). The PCR program consisted of the following: 1 cycle of 95°C for 5 min; 15 cycles of 95°C for 30 s, 70°C for 2 min (−1°C per cycle), and 72°C for 1.5 min; and 15 cycles of 95°C for 30 s, 55°C for 1 min, and 72°C for 1.5 min. PCR product was purified with AMPure XP beads at a ratio of 0.9× and eluted in 15 μl of elution buffer (Qiagen). A total of 0.015 μl of PCR product was amplified with a second round of touchdown PCR using the primers P5_2 plus left- or right-OTC-F2 and the same PCR program as PCR1. PCR product was again purified with AMPure XP beads at a ratio of 0.9× and eluted in 15 μl of elution buffer (Qiagen). DNA libraries were prepared for NGS using unique P7 primers (P701 to P734) for each sample along with P5_2 plus left- or right-OTC-F3 primers and the product of the second PCR as a template. The following PCR program was used for the third and final PCR: 1 cycle of 95°C for 5 min; 15 cycles of 95°C for 30 s, 70°C for 2 min (−1°C per cycle), and 72°C for 30 s; 10 cycles of 95°C for 30 s, 55°C for 1 min, and 72°C for 30 s; and 1 cycle of 72°C for 5 min, 4°C hold. PCR product was purified with AMPure XP beads at a ratio of 2× and eluted in 25 μl of elution buffer (Qiagen). NGS library concentrations were measured by the Quant-It PicoGreen dsDNA assay (Thermo Fisher Scientific, Waltham, MA) before library pooling at equal molarity (*40*). The final pooled and purified NGS library was quantified by a Qubit 3.0 Fluorometer, denatured, and diluted to 8 pM according to Illumina’s instructions with a 15% PhiX spike-in. Sequencing was then performed using custom sequencing primers as described in the GUIDE-seq protocol (*40*) and the Illumina MiSeq Reagent V2 kit 500 cycle to enable successful read merging for downstream data analysis.

Adapter sequences were trimmed using BBduk (https://jgi.doe.gov/data-and-tools/bbtools/). Paired-end read pairs were assembled using PEAR (*41*), and the merged reads were aligned to the reference genomic sequence using BWA-MEM (http://bio-bwa.sourceforge.net/). Unique molecules were identified using UMI-tools (*42*). The molecules were further filtered to ensure they had a mapping quality score ≥20, had a length for the mapped portion ≥60 bp for Hae III–digested samples and ≥55 bp for TaqI-digested samples, and started with the GSP3 primer sequence plus a portion of the homology arm. Unique molecules containing more than 60 bp of aligned portion for Hae III (or 55 bp for TaqI) and less than 10 bp of unaligned portion were classified as “mOTC locus.” For unique molecules containing unaligned portion ≥10 bp, the sequences of unaligned portion were extracted and further aligned to HDR, ITR, SaCas9, polyA, hOTCco, TBG, or other regions on the donor vector and SaCas9 vector sequences using BWA-backtrack (*43*). The HDR integration percentage was calculated as the number of unique molecules mapped to HDR divided by the total number of unique molecules after filtering.

### Statistical analysis

Statistical analyses were performed with GraphPad Prism 6.03 for Windows. The log-rank test was used to test the survival distributions for differences. A one-way analysis of variance (ANOVA) and Dunnett’s multiple comparisons test were used to compare a number of variables with a single control. To compare untargeted and targeted groups, the Mann-Whitney test was used. Because of the relatively small sample size, normality testing was not feasible. Group averages are presented as means ± SEM.

## Supplementary Material

http://advances.sciencemag.org/cgi/content/full/6/7/eaax5701/DC1

Download PDF

A mutation-independent CRISPR-Cas9–mediated gene targeting approach to treat a murine model of ornithine transcarbamylase deficiency

## SUPPLEMENTARY MATERIALS

Supplementary material for this article is available at http://advances.sciencemag.org/cgi/content/full/6/7/eaax5701/DC1 Fig. S1. Schematic diagrams of the OTC locus and the targeted OTC locus by HDR or by NHEJ are shown. Fig. S2. Gene targeting efficiency analysis by ligation-mediated PCR coupled with unique molecular indices (LMU-PCR). Table S1. PCR primer sequences for on-target indel analysis and LMU-PCR.

## Appendix

View/request a protocol for this paper from *Bio-protocol*.
