# Supplementary material for: A mutation-independent CRISPR-Cas9–mediated gene targeting approach to treat a murine model of ornithine transcarbamylase deficiency
Source: Sci Adv. 2020 Feb 12;6(7):eaax5701. doi: 10.1126/sciadv.aax5701 (PMC7015695; doi:10.1126/sciadv.aax5701)
Supplement: http://advances.sciencemag.org/cgi/content/full/6/7/eaax5701/DC1 [file supp_6_7_eaax5701__index.html]

Science Advances | Science AdvancesAAASSearchScience AdvancesMenu

## Supplementary Materials

**This PDF file includes:**

- Fig. S1. Schematic diagrams of the OTC locus and the targeted OTC locus by HDR or by NHEJ are shown.
- Fig. S2. Gene targeting efficiency analysis by ligation-mediated PCR coupled with unique molecular indices (LMU-PCR).
- Table S1. PCR primer sequences for on-target indel analysis and LMU-PCR.

Download PDF

**Files in this Data Supplement:**

- Adobe PDF - aax5701\_SM.pdf
