# Supplementary material for: A mutation-independent CRISPR-Cas9–mediated gene targeting approach to treat a murine model of ornithine transcarbamylase deficiency
Source: Sci Adv. 2020 Feb 12;6(7):eaax5701. doi: 10.1126/sciadv.aax5701 (PMC7015695; doi:10.1126/sciadv.aax5701)
Supplement: Download PDF [file aax5701_SM.pdf]

Science Advances
AAAS

advances.sciencemag.org/cgi/content/full/6/7/eaax5701/DC1

Supplementary Materials for

A mutation-independent CRISPR-Cas9–mediated gene targeting approach to treat a murine model of ornithine transcarbamylase deficiency

Lili Wang, Yang Yang, Camilo Ayala Breton, Peter Bell, Mingyao Li, Jia Zhang, Yan Che, Alexei Saveliev, Zhenning He, John White, Caitlin Latshaw, Chenyu Xu, Deirdre McMenamin, Hongwei Yu, Hiroki Morizono, Mark L. Batshaw, James M. Wilson*

*Corresponding author. Email: wilsonjm@upenn.edu

Published 12 February 2020, *Sci. Adv.* **6**, eaax5701 (2020)
DOI: 10.1126/sciadv.aax5701

This PDF file includes:

Fig. S1. Schematic diagrams of the OTC locus and the targeted OTC locus by HDR or by NHEJ are shown.
Fig. S2. Gene targeting efficiency analysis by ligation-mediated PCR coupled with unique molecular indices (LMU-PCR).
Table S1. PCR primer sequences for on-target indel analysis and LMU-PCR.

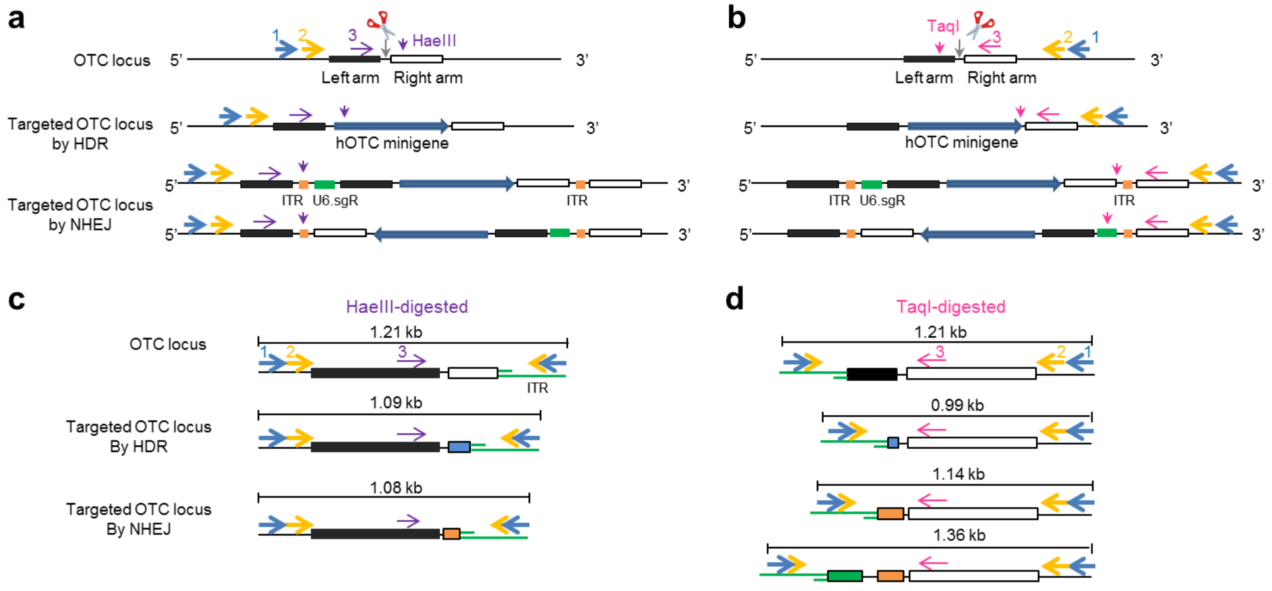

Fig. S1. Schematic diagrams of the OTC locus and the targeted OTC locus by HDR or by NHEJ are shown. Genomic DNA was subjected for restriction enzyme digestion with HaeIII or TaqαI followed by ligation with the linkers containing unique molecular indices. The first two nested PCR reactions were performed using primer pairs hybridizing to the mOTC locus outside the homology arms and the linkers. The third PCR reaction was performed using primer pairs hybridizing to one of the homology arms and the linkers and subjected for deep sequencing. **a**) Diagrams of the OTC locus indicated with the closest HaeIII sites downstream of primer 3. **b**) Diagrams of the OTC locus indicated with the closest TaqαI sites upstream of primer 3. **c**, **d**) Lengths of the first PCR amplicons on HaeIII-digested (**c**) or TaqαI-digested (d) genomic DNA for untargeted and targeted mOTC locus are indicated. The HDR-mediated gene targeting efficiency was determined by the percentage of reads containing the expected hOTCco minigene sequence among the total mapped reads.

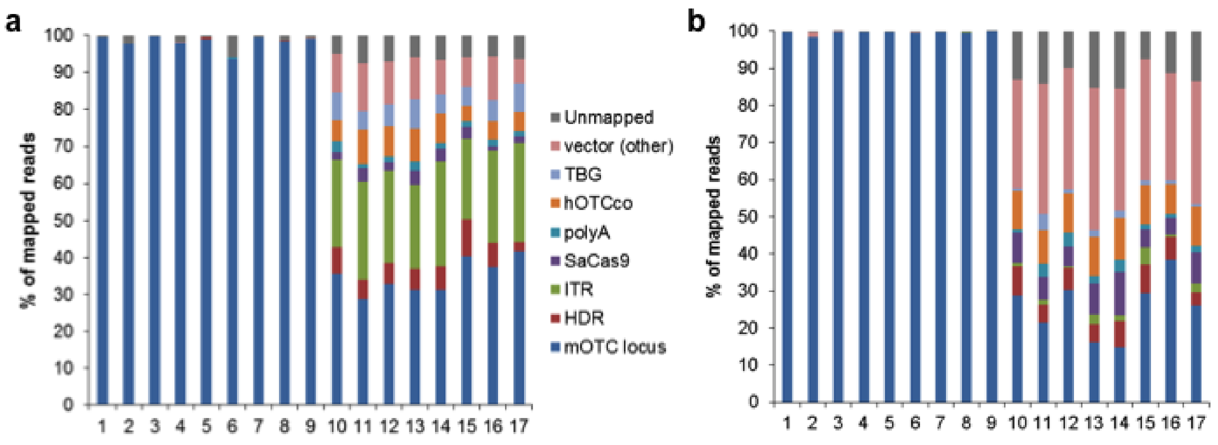

Fig. S2. Gene targeting efficiency analysis by ligation-mediated PCR coupled with unique molecular indices (LMU-PCR). Liver DNA were isolated from *spf*^{ash} mice eight weeks post neonatal treatment with the dual gene targeting vectors (#10-17) or untargeted vectors (#2-9). DNA from an untreated *spf*^{ash} mouse served as control (#1). **a**, **b**) All samples were digested with HaeIII (a) or TaqαI (b), followed by LMU-PCR and deep sequencing. Mapped unique reads were categorized to the mOTC locus, and reads with insertions at the cleavage site were further categorized based on their alignment with the vector sequences to HDR, ITR, SaCas9 cDNA, polyA, and hOTCco cDNA, the TBG promoter, and other parts of vector. Unmapped reads containing inserted DNA sequence could not be aligned to the above segments.

Table S1. PCR primer sequences for on-target indel analysis and LMU-PCR.

Primer	Sequence 5' to 3'
OTC_PointM_F	GGCTATGCTTGGGAATGTCCT
OTC_PointM_R	GCTACAGAATGAAAGAGAGGCG
Left-OTC-F1	GAGTTTAACTGTGGCTTGAGG
Left-OTC-F2	CGGAGTCCTATGTGTCTAGTTC
Left-OTC-F3	CCTCTCTATGGGCAGTCGGTGATAGTCTCACAGACACCGCTC
Right-OTC-F1	TTAGTGTCCAATAAGTGTTAGCTGT
Right-OTC-F2	ACAGATGAGCTTTGACTTACTGAAC
Right-OTC-F3	CCTCTCTATGGGCAGTCGGTGATCCATTATCTAAGGAGAAGCATCATC
P5_1	AATGATACGGCGACCACCGAGATCTA
P5_2	AATGATACGGCGACCACCGAGATCTACAC
p701	CAAGCAGAAGACGGCATACGAGATTCGCCTTAGTGACTGGAGTCCTCTCTATGGGCAGTCGGTGA
p702	CAAGCAGAAGACGGCATACGAGATCTAGTACGGTGACTGGAGTCCTCTCTATGGGCAGTCGGTGA
p703	CAAGCAGAAGACGGCATACGAGATTTCTGCCTGTGACTGGAGTCCTCTCTATGGGCAGTCGGTGA
p704	CAAGCAGAAGACGGCATACGAGATGCTCAGGAGTGACTGGAGTCCTCTCTATGGGCAGTCGGTGA
p705	CAAGCAGAAGACGGCATACGAGATAGGAGTCCGTGACTGGAGTCCTCTCTATGGGCAGTCGGTGA
p706	CAAGCAGAAGACGGCATACGAGATCATGCCTAGTGACTGGAGTCCTCTCTATGGGCAGTCGGTGA
p707	CAAGCAGAAGACGGCATACGAGATGTAGAGAGGTGACTGGAGTCCTCTCTATGGGCAGTCGGTGA
p708	CAAGCAGAAGACGGCATACGAGATCCTCTCTGGTGACTGGAGTCCTCTCTATGGGCAGTCGGTGA
p709	CAAGCAGAAGACGGCATACGAGATTTACCGACGTGACTGGAGTCCTCTCTATGGGCAGTCGGTGA
p710	CAAGCAGAAGACGGCATACGAGATAGTGACCTGTGACTGGAGTCCTCTCTATGGGCAGTCGGTGA
p711	CAAGCAGAAGACGGCATACGAGATTCGGATTCGTGACTGGAGTCCTCTCTATGGGCAGTCGGTGA
p712	CAAGCAGAAGACGGCATACGAGATCAAGGTACGTGACTGGAGTCCTCTCTATGGGCAGTCGGTGA
p713	CAAGCAGAAGACGGCATACGAGATTCCTCATGGTGACTGGAGTCCTCTCTATGGGCAGTCGGTGA
p714	CAAGCAGAAGACGGCATACGAGATGTCAGTCAGTGACTGGAGTCCTCTCTATGGGCAGTCGGTGA
p715	CAAGCAGAAGACGGCATACGAGATCGAATACGGTGACTGGAGTCCTCTCTATGGGCAGTCGGTGA
p716	CAAGCAGAAGACGGCATACGAGATTCTAGGAGGTGACTGGAGTCCTCTCTATGGGCAGTCGGTGA
p717	CAAGCAGAAGACGGCATACGAGATCGCAACTAGTGACTGGAGTCCTCTCTATGGGCAGTCGGTGA
p718	CAAGCAGAAGACGGCATACGAGATCGTATCTCGTGACTGGAGTCCTCTCTATGGGCAGTCGGTGA
p719	CAAGCAGAAGACGGCATACGAGATGTACACCTGTGACTGGAGTCCTCTCTATGGGCAGTCGGTGA
p720	CAAGCAGAAGACGGCATACGAGATCGGCATTAGTGACTGGAGTCCTCTCTATGGGCAGTCGGTGA
p721	CAAGCAGAAGACGGCATACGAGATTCGTCTGAGTGACTGGAGTCCTCTCTATGGGCAGTCGGTGA
p722	CAAGCAGAAGACGGCATACGAGATAGCCTATCGTGACTGGAGTCCTCTCTATGGGCAGTCGGTGA
p723	CAAGCAGAAGACGGCATACGAGATCTGTACCAGTGACTGGAGTCCTCTCTATGGGCAGTCGGTGA
p724	CAAGCAGAAGACGGCATACGAGATAGACCTTGGTGACTGGAGTCCTCTCTATGGGCAGTCGGTGA
p725	CAAGCAGAAGACGGCATACGAGATAGGATAGCGTGACTGGAGTCCTCTCTATGGGCAGTCGGTGA
p726	CAAGCAGAAGACGGCATACGAGATCCTTCCATGTGACTGGAGTCCTCTCTATGGGCAGTCGGTGA
p727	CAAGCAGAAGACGGCATACGAGATGTCCTTGAGTGACTGGAGTCCTCTCTATGGGCAGTCGGTGA
p728	CAAGCAGAAGACGGCATACGAGATTGCGTAACGTGACTGGAGTCCTCTCTATGGGCAGTCGGTGA
p729	CAAGCAGAAGACGGCATACGAGATCACAGACTGTGACTGGAGTCCTCTCTATGGGCAGTCGGTGA
p730	CAAGCAGAAGACGGCATACGAGATTTACGTGCGTGACTGGAGTCCTCTCTATGGGCAGTCGGTGA
p731	CAAGCAGAAGACGGCATACGAGATCCAAGGTTGTGACTGGAGTCCTCTCTATGGGCAGTCGGTGA
p732	CAAGCAGAAGACGGCATACGAGATCACGCAATGTGACTGGAGTCCTCTCTATGGGCAGTCGGTGA
p733	CAAGCAGAAGACGGCATACGAGATTTCCAGGTGTGACTGGAGTCCTCTCTATGGGCAGTCGGTGA

p734	CAAGCAGAAGACGGCATACGAGATTCATCTCCGTGACTGGAGTCCTCTCTATGGGCAGTCGGTGA
A01	AATGATACGGCGACCACCGAGATCTACACTAGATCGCNNWNNWNNACACTCTTTCCCTACACGACGCTCTTCCGATCT
A02	AATGATACGGCGACCACCGAGATCTACACCTCTCTATNNWNNWNNACACTCTTTCCCTACACGACGCTCTTCCGATCT
A03	AATGATACGGCGACCACCGAGATCTACACTATCCTCTNNWNNWNNACACTCTTTCCCTACACGACGCTCTTCCGATCT
A04	AATGATACGGCGACCACCGAGATCTACACAGAGTAGANNWNNWNNACACTCTTTCCCTACACGACGCTCTTCCGATCT
A05	AATGATACGGCGACCACCGAGATCTACACGTAAGGAGNNWNNWNNACACTCTTTCCCTACACGACGCTCTTCCGATCT
A06	AATGATACGGCGACCACCGAGATCTACACACTGCATANNWNNWNNACACTCTTTCCCTACACGACGCTCTTCCGATCT
A07	AATGATACGGCGACCACCGAGATCTACACAAGGAGTANNWNNWNNACACTCTTTCCCTACACGACGCTCTTCCGATCT
A08	AATGATACGGCGACCACCGAGATCTACACCTAAGCCTNNWNNWNNACACTCTTTCCCTACACGACGCTCTTCCGATCT
A09	AATGATACGGCGACCACCGAGATCTACACGACATTGTNNWNNWNNACACTCTTTCCCTACACGACGCTCTTCCGATCT
A10	AATGATACGGCGACCACCGAGATCTACACACTGATGGNNWNNWNNACACTCTTTCCCTACACGACGCTCTTCCGATCT
A11	AATGATACGGCGACCACCGAGATCTACACGTACCTAGNNWNNWNNACACTCTTTCCCTACACGACGCTCTTCCGATCT
A12	AATGATACGGCGACCACCGAGATCTACACCAGAGCTANNWNNWNNACACTCTTTCCCTACACGACGCTCTTCCGATCT
A13	AATGATACGGCGACCACCGAGATCTACACCATAGTGANNWNNWNNACACTCTTTCCCTACACGACGCTCTTCCGATCT
A14	AATGATACGGCGACCACCGAGATCTACACTACCTAGTNNWNNWNNACACTCTTTCCCTACACGACGCTCTTCCGATCT
A15	AATGATACGGCGACCACCGAGATCTACACCGCGATATNNWNNWNNACACTCTTTCCCTACACGACGCTCTTCCGATCT
A16	AATGATACGGCGACCACCGAGATCTACACTGGATTGTNNWNNWNNACACTCTTTCCCTACACGACGCTCTTCCGATCT
